# Salvianolic acid A & B: potential cytotoxic polyphenols in battle against cancer via targeting multiple signaling pathways

**DOI:** 10.7150/ijbs.37467

**Published:** 2019-08-19

**Authors:** Tian Qin, Azhar Rasul, Ayesha Sarfraz, Iqra Sarfraz, Ghulam Hussain, Haseeb Anwar, Ammara Riaz, Sitong Liu, Wei Wei, Jiang Li, Xiaomeng Li

**Affiliations:** 1 The Key Laboratory of Molecular Epigenetics of MOE, Institute of Genetics and Cytology, Northeast Normal University, Changchun 130024, China; 2 Department of Zoology, Faculty of Life Sciences, Government College University Faisalabad, 38000, Faisalabad, Pakistan; 3 Neurochemicalbiology and Genetics Laboratory (NGL), Department of Physiology, Faculty of Life Sciences, Government College University Faisalabad, 38000, Faisalabad, Pakistan; 4 Dental Hospital, Jilin University, Changchun 130021, China; 5 College of Life Sciences, Jilin University, Changchun, 130012, China

**Keywords:** Salvianolic acids, natural products, anticancer activity

## Abstract

Nature has generously offered life-saving therapies to mankind by providing evolutionarily optimized drug-like entities in the form of natural products. These splendid gifts of nature have served as most suitable candidates for anti-cancer drug discovery due to their pleiotropic activity on target molecules. This review aims to provide an update on the natural sources and bioactivities of such gifts from nature, salvianolic acid A & B, which are major bioactive constituents of a traditional Chinses medicinal herb, *Salvia miltiorrhiza*. Salvianolic acid A & B have been reported to owe anti-cancer, anti-inflammatory and cardioprotective activities. Currently salvianolic acids have been emerged as potent anti-cancer molecules. Salvianolic acid A & B fight cancer progression by prompting apoptosis, halting cell cycle and adjourning metastasis by targeting multiple deregulated signaling networks of cancer. Moreover, salvianolic acid A & B display potency towards sensitizing cancer cells to chemo-drugs. The review purposes that salvianolic acid A & B supply a novel opportunity for drug discovery but further experimentation is mandatory to embellish the knowledge of their pharmacological usage and to access their toxicological limits in order to establish these compounds as potential multitarget future drugs.

## Introduction

Medicinal plants and herbs have established their worth as a primary source of bioactive molecules having therapeutic potentiality since times 1. Although synthetic medicine have gained popularity due to their easy quality control, production cost, time effectiveness, quick effects, and tringent regulation, however, efficacy and safety of synthetic medicines was always questionable, eventuating in ultimate dependence of populations on nature-derived products for primary healthcare around the globe 2. Around 80% of human populations are still dependent upon medicinal plants for their health care services 3.

Medicinal plants laid the foundation of first sophisticated medicinal system in Mesopotamia back in 2600 BC 3. Out of 1453 novel small molecules approved by FDA in 2013, 40% are nature inspired compounds either naturally obtained or their derivatives. Approximately 50% of the drugs approved in the past 30 years are derived from natural products 4. Well-known natural products that have been become eminent in present day pharmacotherapy as anti-tumor agents include paclitaxel and its derivatives that are obtained from Taxus species 1. A novel antimalarial agent, Artemisinin, was also firstly isolated from *Artemisia annua* 5.

Various investigations have reported emerging role of natural products as anti-oxidant 6, anti-bacterial 7, anti-inflammatory 8, anti-cancer 9 and neuroprotective agents 10.

Polyphenols are a broad family of natural products encompassing flavonoids 11, tannins, stilbenoids and phenolic acids which are abundantly found in medicinal herbs and plants 12. Since times, polyphenols have been subject of extensive scientific interest due to their possible worthwhile effects on human health 13. Salvianolic acid A and B are stilbenoid and polypropanoid polyphenols which are isolated from radix of* Salvia miltiorrhiza.* Salvianolic acid A and B have been known to possess various bioactivities such as anti-inflammatory 14, anti-cancer 15 and anti-oxidant 16.

Although several researchers have reviewed the role of salvianolic acids as cardio-protective and antioxidant agents but no one has still attempted to review the anti-cancer properties of salvianolic acid. Therefore, this review is an effort to update research community about the anti-cancer potential of salvianolic acids with specifically focusing on their action mechanism. Manual searches were checked by various sites as PubMed, Elsevier and Google. Following keywords were used for searching: Salvianolic acid, natural products, anticancer activity and salvianolic acid A, B and its biological activities.

### Natural sources of salvianolic acid A and B

**Figure 1:**
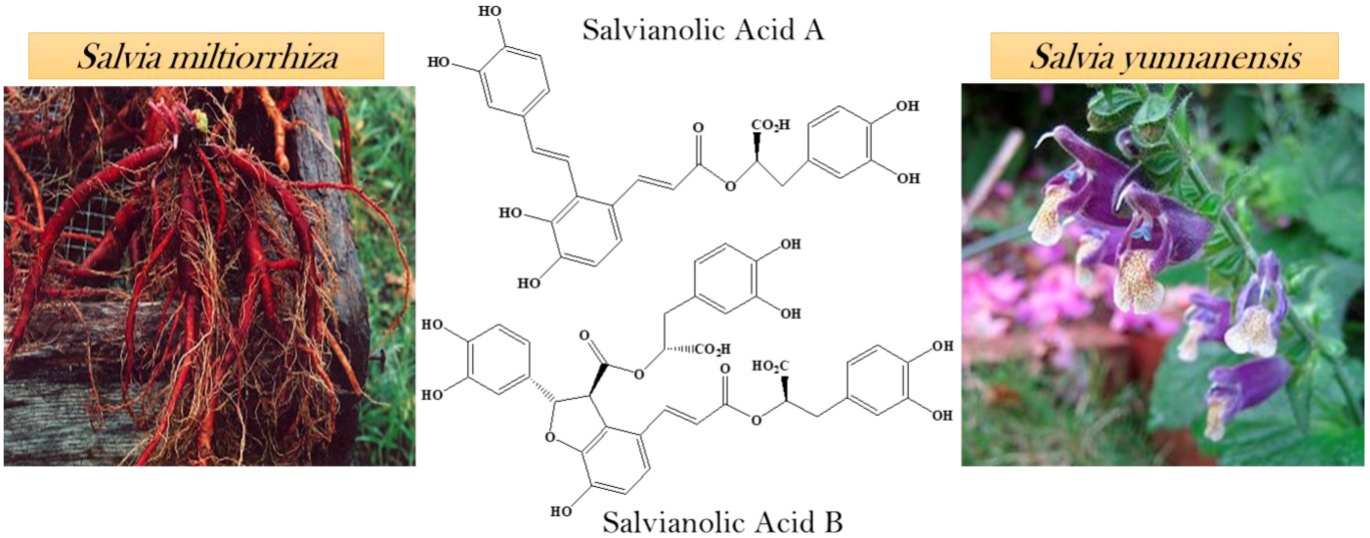
Natural sources of salvianolic acid A & B.

Salvianolic acids are the most abundant compounds of *Salvia miltiorrhiza* which is a Chinese herbal plant. The roots of *Salvia miltiorrhiza* are utilized in Chinese medicines which are extensively used for the cure of cancer. Salvianolic acid A and B (SAA, SAB respectively) has been extracted from the roots of *Salvia miltiorrhiza* 17. From the aqueous root extract of *Salvia yunnanensis*, two known polyphenols were isolated salvianolic acid A and lithospermic acid 18. *Salvia yunnanensis* is also a traditional Chinese herb used as a substitute of *Salvia miltiorrhiza* 19 (Figure 1).

### Biological activities of salvianolic acid A and B

The nature-derived pharmacologically active polyphenolic compounds, salvianolic acids, have been declared to possess various bioactivities such as antiinflammatory, anticancer, antioxidant and cardio-protective. Multiple *in vitro*/ *in vivo* studies have revealed the potential of salvianolic acids as potent anticancer agents.

### Anticancer activity

Cancer is a multifaceted disease characterized by unrestricted cellular proliferation caused due to functional dysregulation of various important genes encoding for key proteins such as tumor suppressers, anti-apoptotic proteins as well as growth factors 20. Treatment of cancer is currently based on chemotherapy which has limited therapeutic success because of high expenses, toxicity and development of resistance 21. Cancer chemoprevention by nature-derived bioactive compounds is now gaining attention because they have the ability to overcome the limitations of the drugs used today 22. Most of the pharmaceutic drugs act as monotarget entities but these multitargeted natural compounds have the ability to regulate proliferation and cancer growth via targeting multiple signaling cascades 22.

Approximately 60% of anticancer agents have been emerged from nature including marine biota, microorganisms and plants 23. Phytochemicals acquired from herbs, fruits, vegetables and medicinal plants such as flavonoids, phenolic compounds and terpenoids have shown promising effects in overcoming carcinogenesis 24.

**Figure 2:**
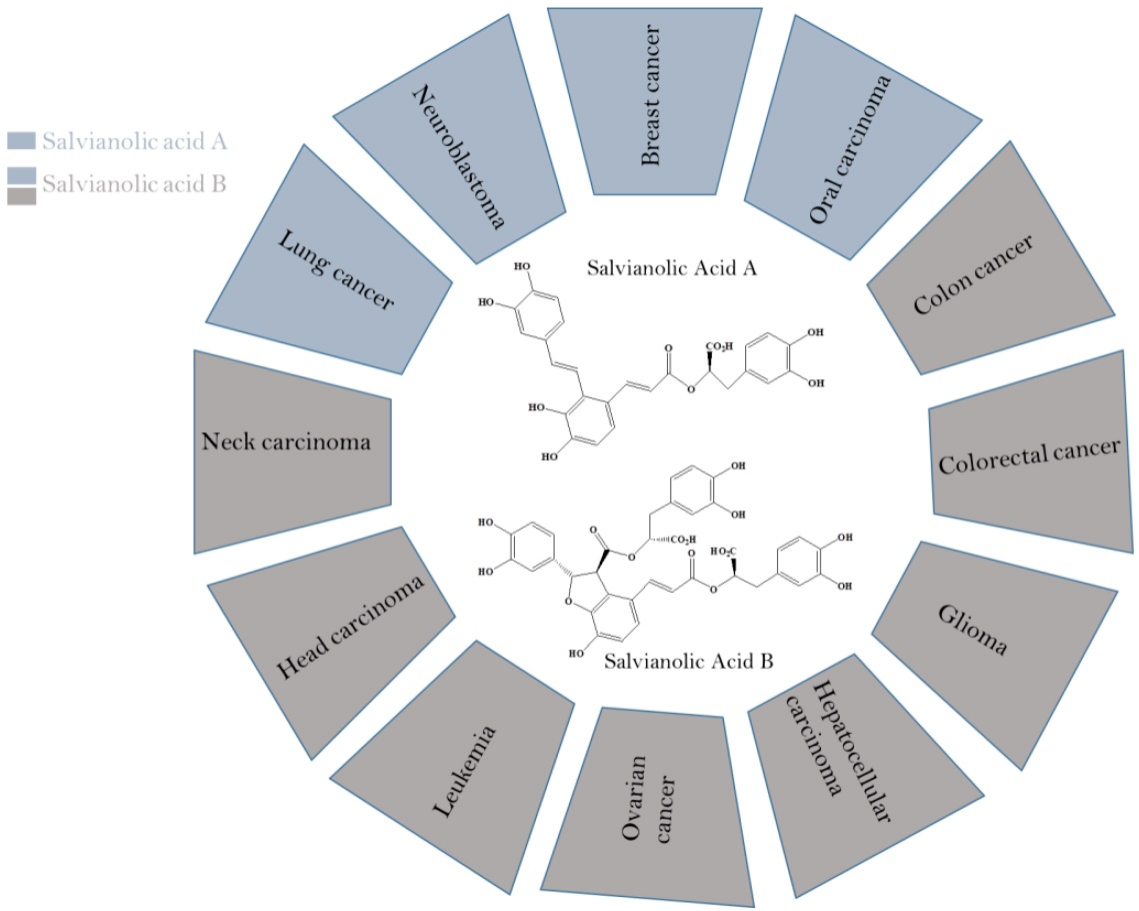
Anti-cancer potential of salvianolic acid A & B against various cancers.

Secondary metabolites isolated from plants as stilbenoids, flavonoids and phenolics have been reported for their potential anticancer activities 25, 26. Stilbenoids and polypropanoid polyphenols have been well-known to owe various health promoting characteristics such as anti-oxidant, anti-tumor, cardio-protective, neuroprotective and antiinflammatory. Salvianolic acid A & B, stilbenoid and polypropanoid polyphenols, have been affirmed to possess antiproliferative properties against A549/PC9 (lung carcinoma) 27, MCF-7 (Breast cancer) 28, SCC-9/SCC-25 (Oral squamous cell carcinoma) 29, HCT-116/HT29 (Colorectal cancer) 30, HN-13/JHU-06 (Head and neck carcinoma) 31, SKOV3 (Ovarian cancer), HepG2/Bel-7404 (Hepatocellular cancer) 32, and U87/U373 (Glioma) cancer cell lines 33 (Figure 2).

### Salvianolic acid A, B and apoptosis

Apoptosis is characterized as regulated and systematized mode of cellular death involving the genetically determined eradication of unwanted cells 34, 35. Apoptosis is considered vital for several intricate biological functions such as embryonic development, immune-system activity and chemical induced cellular death 34. Disruption of this highly regulated process is novel acquired capability of cancerous cells. Reviving the normal apoptotic process is one of the emerging challenges of cancer research 36.

SAA and SAB have been emerged as novel anticancer paradigms for multitargeted prevention of cancer. Anticancer characteristics of SAA and SAB has been revealed to be associated with triggering apoptosis through activation of caspases, reducing anti-apoptotic proteins (Bcl-2), activation of proapoptotic proteins (Bak, Bax), modulating PI3K/ Akt/ MAPK pathways, NF-ĸB inhibition and ROS accumulation (Table 1).

### Targeting intrinsic apoptotic machineries by salvianolic acid A & B

Cells have evolved two main pathways for apoptosis; extrinsic or death receptor pathway and intrinsic pathway 55. These cascades eventually activate the caspases which successively trigger effector caspases. Regulation of Bcl-2 family results in loss of MMP allowing the discharge of cytochrome c through mitochondrial porin channels which stimulate the cascade of caspases that lead towards cell death 56.

**Figure 3:**
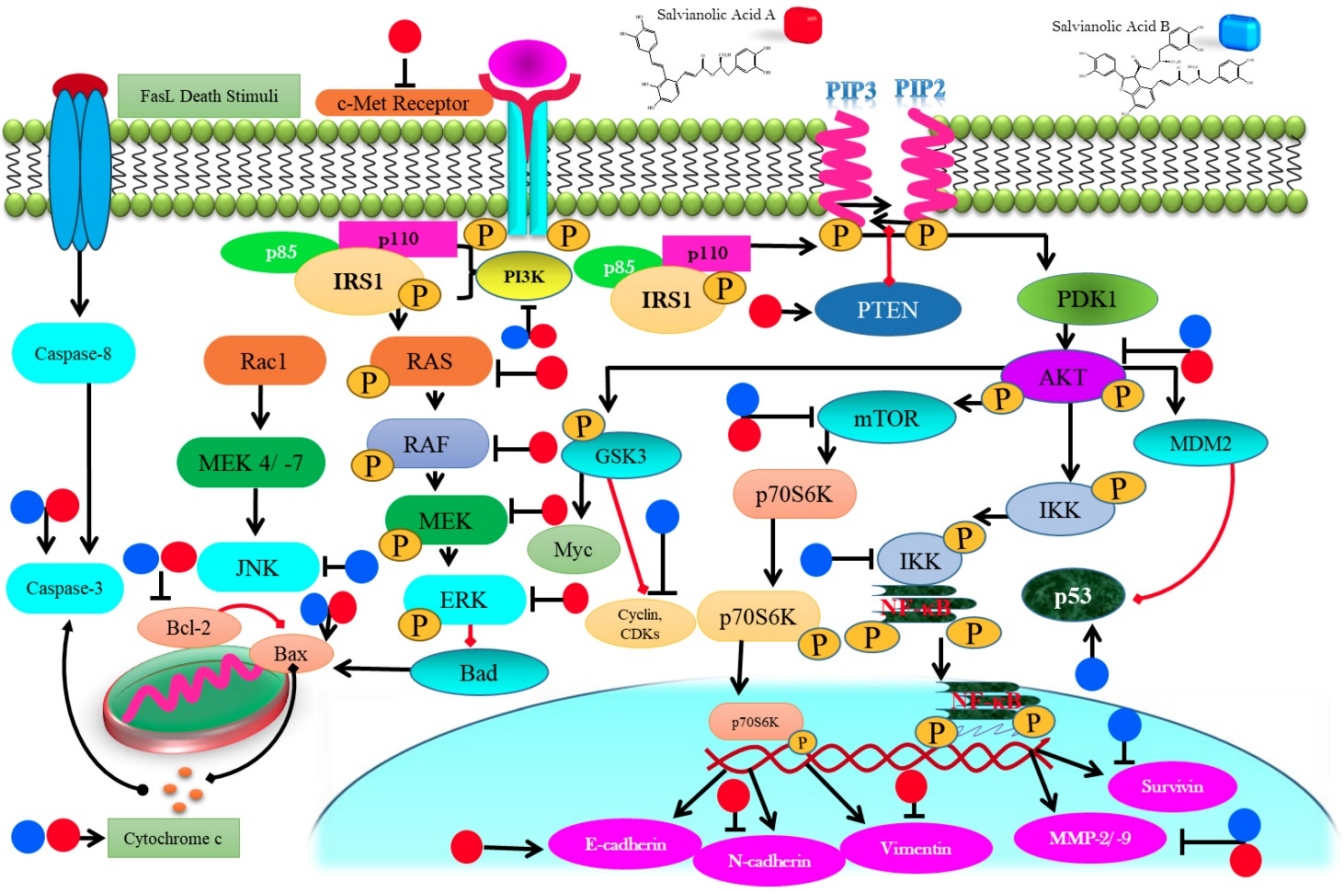
Diagrammatic representation of membrane, cytoplasmic and nuclear targets of salvianolic acid A & B eventuating in anticancer properties in various cancer types.

SAA treatment induced apoptosis in KG-1, THP-1 and Kasumi-1 cells via intrinsic apoptotic pathway by upregulating Bak, decreasing Bcl-xl, followed by cleavage of PARP and caspase-3 activation 41. SAA induced intrinsic apoptosis in resistant MCF-7 cells via Bax up-regulation, lowering Bcl-2, reducing ΔΨ, and activating caspases 28. Treatment of SAB activated caspase 9 which in turn activated caspase-3 to induce apoptosis in HCT116 and HT29 cells 30. SAB treatment enhanced p53 and decreased Bcl-2 in JHU-22 cells, thus, induced apoptosis 46. SAB decreased Bcl-2 and Bcl-xL and enhanced p53 and caspase 3 with cleavage of PARP to induce apoptosis in JHU 013 cells 45. SAB induced caspase-3 mediated apoptosis while reducing livin expression in dose-dependent mode in SKOV3 cells 49 (Figure 3). Although several studies have reported that SAA and SAB mediate intrinsic apoptotic pathway, however, no one has still attempted to explore that whether SAA and SAB could induce apoptosis via extrinsic pathway which calls for further research to shed light on this matter.

### Targeting P13K/Akt and MAPK pathways by salvianolic acid A & B

PTEN, a widely mutated tumor suppressor gene, modulates several intricate cellular networks such as MAPK and PI3K/ Akt pathways, thus, maintaining cellular homeostasis of growth and development 57. Mitogen activated protein kinase (MAPK) encompasses four key signaling domains; Big MAP kinase-1, MAPK/ERK family, c-Jun N terminal kinase (JNK), and p38 MAPK. When these kinases are activated they transmit extracellular cues that regulate apoptosis, proliferation, differentiation and migration of cells 58, 59. The phosphatidylinositol 3-kinase (P13K)/Akt signaling cascade is widely deregulated network in cancer and, therefore, considered an important anti-tumor target 60. Aberrant activation of these pathways is associated with cancer development. Therefore, targeting P13K/Akt and MAPK pathways is an auspicious approach towards cancer treatment 59, 60.

SAA treatment to A549 cells up-regulated p-PTEN in a time-dependent mode. Increased phosphorylation of PTEN significantly suppressed PI3K/AKT signaling, thus, reducing invasive capabilities of cancerous cells 38. SAA inhibited the expression of p-RAF, p-MEK1/2 as well as p-ERK1/2 which controlled MMP-2 level in SCC-25 and SCC-9 cells. These molecular events led to the markedly reduced metastatic abilities of cancer cells 42. SAA decreased the p-AKT expression levels, thus, reducing proliferation of in KG-1, THP-1 and Kasumi-1leukemia cells *in vitro* 41. SAB prompted autophagic cell death in HT29, HT116 and SK-Hep-1 via decreasing phosphorylated Akt and its down-stream target mTOR. Reduced phosphorylation of mTOR also failed to phosphorylate p70S6K which recommend efficacy of SAB as potent inhibitor of Akt/mTOR pathway 30, 50. SAB induced the phosphorylation of p38 MAPK and p53 in a dose-mediated mode in U87 which reduced proliferation of cancer cells 33 (Figure 3). Though various investigations has reported that SAA and SAB target PI3K/ Akt pathway but role of SAA and SAB in mediating various other downstream targets of PI3K/Akt pathway such as GSK3 and FOXOs should also be investigated in future studies.

### Targeting NF-κB pathway by salvianolic acid A & B

NF-κB, a downstream target of STAT3, is a family of five transcriptional factors (NF-κB1/p105, NF-κB2/p100, c-Rel, RelB and RelA/p65) which form specific protein complexes and via binding at the promoter region of DNA, this family regulates a number of essential cellular processes 61. Constitutive activation of NF-κB is responsible for stimulating cellular proliferation, preventing apoptosis, regulating angiogenesis, promoting metastasis, and remolding tumor metabolism by induction of various genetic expressions such as COX-2 62, 63. Thus, NF-κB is principle factor which control the capability of malignant and pre-neoplastic cells to resist against apoptosis and encourage tumor invasion, proliferation and angiogenesis. Therefore, targeting NF-κB could serve as a tempting avenue to combat cancer and halt metastasis 64.

SAB lowered the levels of NF-κB and MDM-2 and caused dose-mediated blockage of LPS stimulated PGE2 and COX-2 in treated JHU-013 cells 45. SAB sequentially inhibited the COX-2/PGE2/EGFR signaling pathway in JHU-13 cells 46. Although NF-κB targeting has been reported by SAB but whether it directly targets NF-κB or via its upstream pathways Akt/ JAK or STAT3 should be investigated. Moreover, SAB has been reported as safe therapeutic entity for cardiovascular diseases, thus, it would have much more potential as anticancer agent because COX-2 inhibitors such as celecoxib are frequently reported to impose cardiovascular toxicity. Therefore, in the light of these observations it is purposed that SAB could presents its dual efficacy as chemotherapeutic as well as cardio-protective agent which calls for further in depth research to turn SAB into a potent drug lead.

### Halting metastasis by salvianolic acid A & B

Metastasis remains the most complicated hurdle to overcome for cancer treatment that accounts for 90% of the cancer related deaths 65. Epithelial to mesenchymal transitions (EMT) is the ultimate requisite of invasiveness which is mediated by various cell adhesion entities, intermediate filaments such as E-cadherin, N-cadherin, and vimentin 66, 67. Moreover, matrix metalloproteinase such as MMP-2/ -9 are indispensable to derive the metastasis of cancer cells 68, 69. Thus, targeting these imperative mediators of metastasis has potential to halt metastasis and overcome invasiveness of cancerous cells.

**Figure 4:**
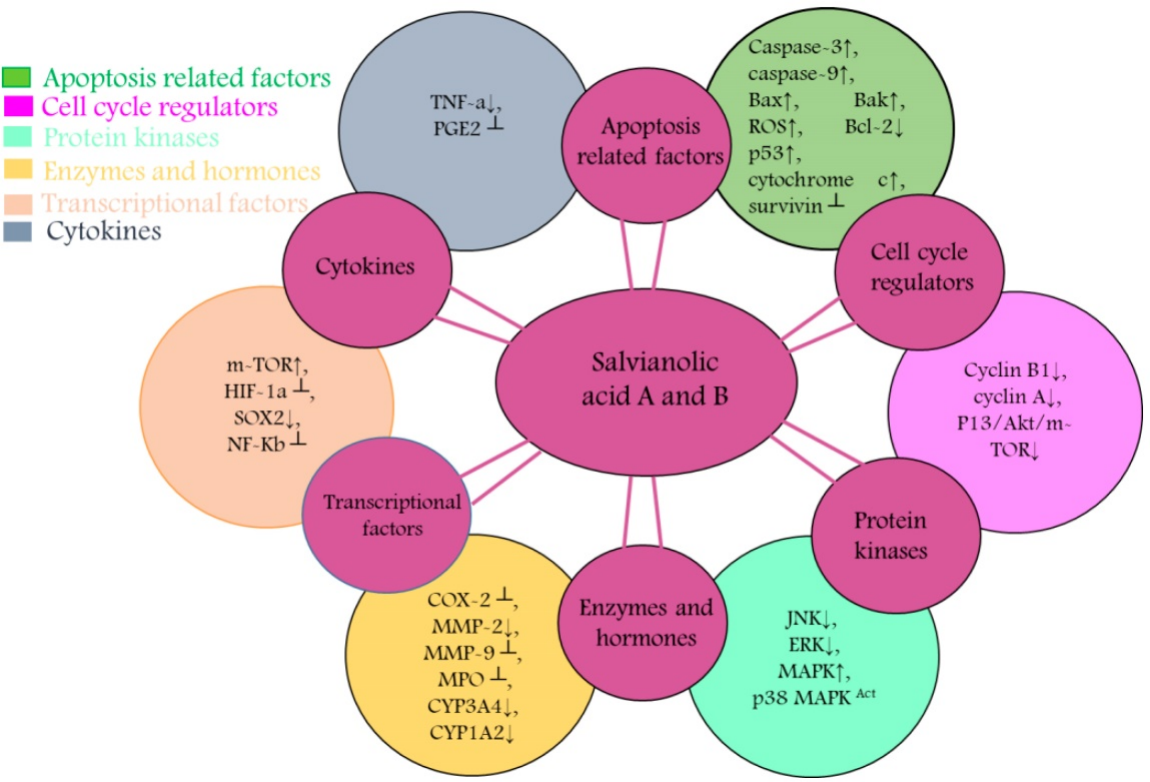
Diagrammatic representation of mechanism of action of Salvianolic A & B resulting in chemopreventive and chemotherapeutic activity. These entities targets and cause activity modulation of various protein kinases, transcriptional factors, apoptosis related factors, cytokines, cell cycle regulators, enzymes and hormones that are associated with proliferation, metastasis, invasion and angiogenesis.

SAA blocked EMT via enhancing E-cadherin (epithelial marker) while significantly lowering the levels of vimentin and N-cadherin, thus, inhibiting the migration and invasive capabilities of MCF-7 cells 39. SAA reduced MMP-2 expression in a Raf/MEK/ERK mediated manner in SCC-9 cells to control migration of cells 42 (Figure 4). Though SAA and SAB have been reported to halt metastasis via targeting EMT markers such as E-cadherin but additional investigations should also explore the effect of SAA and SAB to other EMT markers such as ZEB-1, ZEB-2 and TCF3.

### Conquering drug resistance by salvianolic acid A & B

Emergence of resistance against chemo-drugs is a major challenge towards effective cancer treatment. Thus, therapies overcoming drug resistance are greatly needed 70. c-MET is a tyrosine kinase receptor which is activated by ligand HGF (hepatocyte growth factor). Upon activation, via interplay with other tyrosine kinases, it has potential to stimulate several downstream signaling networks such as PI3K/Akt, mTOR, MAPK, and Ras/Raf/Erk. All these activated pathways provide multiple advantages to cancer cells such as invasion, metastasis and resistance against anti-cancer agents. Thus, targeting c-MET could help to overcome the biggest challenge of drug resistance 71, 72.

Salvianolic acid A has potential to attenuate Akt/mTOR network via blocking c-MET expression which ultimately restores the sensitivity of A549/DDP cells towards cisplatin. Combined treatment of SAA and cisplatin significantly lowered the IC_50_ values of cisplatin towards resistant lung cancer cells 27. SAA reversed paclitaxel resistance in MCF-7 cells and sensitized cancer cells to paclitaxel 10 folds at 12 μM concentration. SAA significantly prevented the expression of transgelin 2 and ABC transporters such as MRP1, P-gp and BCRP and caused effective inhibition of PI3K/Akt signaling in MCF-7 cells to drive the sensitivity of cancer cells towards chemo-drugs 40. Salvianolic acids markedly suppressed P-gp, protein associated with resistance, which was mediated by ROS generation in /MDR MCF-7 and HCT‑8 cells. As ROS induction has potential to sensitize cancer cells to chemo-drugs 28, 43.

### *In vivo* studies and biosafety profile

Administration of 5 and 20 mg/ kg SAA to leukemia xenografted mouse model significantly inhibited growth of tumors. Treatment of SAA didn't induce any alterations in body weight and overall health of the treated animals suggesting that SAA might turn up as safer chemotherapeutic agent. Moreover, SAA treatment to primary leukemia cells obtained from acute myeloid leukemia patients induced apoptosis in 80-95% of the cells at the concentration of 50-100 μM 41. Comparison of SAA and doxorubicin treatment to MCF-7/MDR xenografted model declared that SAA has potency to significantly reduce tumor weight. Furthermore, SAA treatment didn't affect the weight of tumor bearing mouse which in the case of doxorubicin treatment was markedly reduced which suggests lower toxicity and efficacy of SAA 28. SAB reversed the multi drug resistance in colon CSCs (cancer stem cells) xenografted mouse model via reduction of SOX2, CD44 and ABCG2 expression 73. Combined treatment of SAB (80 mg/kg) and celecoxib (2.5 mg/kg) for 24 days via intraperitoneal injection significantly inhibited growth of tumors JHU-013 xenografted model 46. SAB reduced the volume of tumors significantly in U87 xenografted model when administered intravenously at the dose of 80 mg/kg 33. SAB has potential to attenuate the enhanced glycolysis and glutaminolysis while reduced myoinositol and cholesterol metabolism in DMBA induced hamster model to normal state. Moreover, SAB inhibited key regulator of angiogenesis such as HIF-1α, MMP9 and TNFα which ultimately reduced the proliferation of cancer cells 29, 51.

To assess the biosafety profile and toxicity of SAB in rats, combinatorial treatment of SAB and ginsenoside Rg1 was given to mice. LD_50_ value was 1747 mg/kg, which is hundred times higher than its effective dose. Moreover, no toxicity to heart, brain, kidney, lung and liver structure was observed at any dose which clearly represent that SAB is a bio safe natural entity 74. A number of clinical trials have declared the efficacy of *S. miltiorrhiza* for the treatment of stroke, heart attack and several other pathological conditions 75. Being bioactive constituents of a bio safe traditional medicine utilized from 100 of years, salvianolic acids might emerge as safer chemotherapeutic agents along with their chemopreventive potential. Thus, further *in vivo*, pharmacodynamic and pharmacokinetic studies along with preclinical trials are clearly needed to establish these compounds as potent natural cancer killers in future.

## Conclusion and future directions

In this article, we emphasized on the journey of salvianolic acid A and B as potent anticancer and anti-tumor agent. Organized data from multiple lines of evidences have provided a clearer image of the effective role of SAA and SAB against different cancer types. SAA and SAB have broad range of toxicity against numerous human cancerous cells. Salvianolic acids mechanistically proceed through modulation of various signaling networks such as MAPK, P13K/Akt, NF-κB and mTOR and pathways which are often deregulated in cancers and are also associated with drug resistance. In the light of these reports, it can be hypothesized that SAA and SAB may become potent lead compounds for anticancer drugs but additional experimentation, preclinical trials and medicinal chemistry studies are yet required to explore full spectrum of its pharmaceutic potential. Its effectiveness and safety could offer much more commercial value for medicinal purposes, thus, the next drug lead might be just around the corner and are we ready to pursue this opportunity.

## Figures and Tables

**Table 1:** Molecular targets of Salvianolic acid A & B against numerous cancers

Cancer type	Cell line	No. of cells/well	Treatment time	EC_50_	Molecular targets	Cell cycle arrest	References
Salvianolic acid A							
Lung	A549, H1975, PC9	5x10, 3x10, 1x10^4^	24 h	11.12 μM	c-Met↓, Akt/mTOR↓, MDR1↓, p-Akt↓, m-TOR↑, p-PTEN↑, caspase-3↑	G1	27, 37, 38
Breast	MCF-7	5000 5x10^5^/ml	24 h	98.9 μmol/l	ROS↑, caspase-3↑, Bcl-2↓, Bax↑, p-gp↓, Transgelin 2↓, P13K/Akt┴ , BCRP↑, E-cadherin↑, N-cadherin↓, Vimentin↓, ΔΨm↓	S	28, 39, 40
Leukemia	THP-1, KG-1, Kasumi-1	1.5x10^5^	6, 12, 24 h	5.86 μM, 42.55 μM, 39.16 μM	Bak↑, Bcl-xl↓, caspase-3^Act^, P13/Akt↓, Bcl-2↓,	--	41
Oral	SCC-9, SCC-25	1x10^5^	24 h	--	p-c-Raf┴, p-ERK1/2┴, p-MEK1/2┴, MMP-2↓	--	42
Salvianolic acid B							
Colorectal	HCT116, HT29, HCT8	5x10^3^	--	--	Akt/mTOR↓, Bcl-2↓, Bax↑, ROS↑, p-gp↓	--	30, 43
Head and neck	HN13, HN30, JHU-06, JHU-013	3x10^3^, 1x10^4^	24 h	18 μM, 50 μM	NF-ĸB┴, TNF-a↓, JNK↓, ERK↓, COX-2┴, Prostaglandins E2┴	G0/ G1	31, 44-46
Breast	MCF-7, MDA-MB-231	1 x 10^4^	24 h	--	CyclinB1↓, Cyclin A↓, Bcl-xL┴, Survivin┴, p-ERK↓	--	47, 48
Ovarian	SKOV3	--	--	45.6 μmol/L	Livin↓, Caspase-3↑	G0/ G1	49
Liver	SK-Hep-1, Bel-7404, HepG2 HL-7702	3x10^3^	24 h	143.8 μM, 240.11 μM 758.63 μM	Akt/mTOR┴, Caspase3↑, Caspase-9↑, Cytochrome c↑, LC3-II↑, P62↓, CYP3A4↓, CYP1A2↓	--	32, 50
Oral	CAL27, SCC4	1x10^3^	24, 48, 72 h	51 g/ml, 87 g/ml	HIF-1α ┴, TNF-α ┴, MMP9┴, THBS2↑, VEGF↑	--	29, 51, 52
Brain	U87, U373	1x10^4^	24 h	--	Fis-1↓, ROS↑, p38MAPK↑, p53↑ ROS↑, Bax/Bcl-2↑, Caspase-3 ^Act^, MMP┴	--	33, 53
Lung	A549	1 x 10^5^	--	255.1 μM, 48.4 μM	COX-2┴	--	54

c-MET: mesenchymal-epithelial transition factor; PI3K: phosphatidylinositol-3 kinase; mTOR: mammalian target of rapamycin; Bcl-2: B-cell lymphoma 2; transcription factor sox-2: SOX2; Bax: Bcl2-associated X protein; ATP-binding cassette sub-family G member 2: ABCG2; MAPK: mitogen activated protein kinase; MMP: matrix metalloproteinase; COX-2: cyclooxygenase-2; cluster of differentiation: (CD)44; MRP1: multidrug resistance-associated protein 1; P-gp: P-glycoprotein; BCRP: breast cancer resistance protein; NF-κB: Nuclear factor kappa B.
